# Supplementary material for: Structural invariants and semantic fingerprints in the “ego network” of words
Source: PLoS One. 2022 Nov 22;17(11):e0277182. doi: 10.1371/journal.pone.0277182 (PMC9681103; doi:10.1371/journal.pone.0277182)
Supplement: S1 Appendix — In this appendix we provide additional information regarding the data preprocessing, the soft clustering analysis, and we include additional tables to support the findings in the paper. (PDF) [file pone.0277182.s001.pdf]

S1 Supporting information

S1.1 Data preprocessing: filtering out inactive Twitter users

In order to be relevant to our work, a Twitter account must be an active account, which we define as an account not abandoned by its user and that tweets regularly. A Twitter account is considered abandoned, and we discard it, if the time since the last tweet is significantly bigger (we set this threshold at 6 months, as previously done also in [1]) than the largest period of inactivity for the account. We also consider the tweeting regularity, measured by counting the number of months where the user has been inactive. The account is tagged as sporadic, and discarded, if this number of months represents more than 50% of the observation period (defined as the time between the first tweet of a user in our dataset and the download time). We also discard accounts whose entire timeline is covered by the 3200 tweets that we are able to download, because their Twitter behaviour might have yet to stabilise (it is known that the tweeting activity needs a few months after an account is created to stabilise).

S1.2 Ruling out soft clustering for the creation of semantic profiles

In discussed in the body of the paper, the hard clustering approach to topic extraction yields many unassigned words (Table 5). We have thus also tested soft clustering, where by each word occurrence is assigned, in any case, a probability distribution of belonging to one of the 100 topics. In Fig S1 we plot the fraction of the semantic profile covered by the top-x topics in the ring (where top-x is computed based on the semantic profile $P_r^{(e)}$). Unlike hard clustering, soft clustering gives non-zero values to the least important topics of the ring. While

soft clustering allows us to include all tweets in our analysis, it has a very negative side effect. As we show in the following of the section, very generic topics become prevalent, and mask more characteristic topics that hard clustering reveals, particularly for the innermost rings. Notice that this side effect makes all rings look alike in terms of number of active topics, as we can see from the fact that all distribution curves overlap in the right-hand side plots of Fig S1.

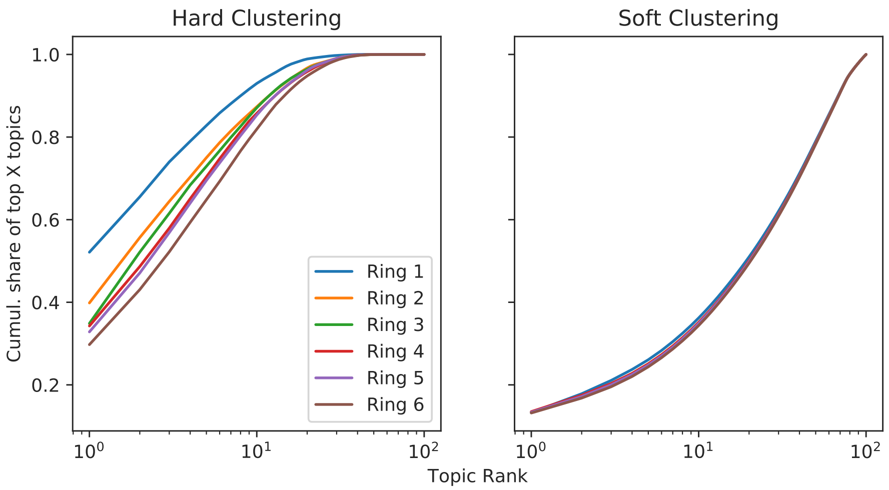

Fig. S1. Hard vs soft clustering. Fraction of the semantic profile covered by the top-x topics in the ring, after hard (left) and soft (right) clustering.

To better investigate this aspect, we extract the important topics as described in Section 5.2.3. With two classes (important vs non-important), we obtain an average silhouette score of 0.9, confirming the good cluster configuration. We show these results for the Journalists dataset but similar conclusions can be drawn for the others. In Fig S2, we compare the level of importance of the 5 most dominant topics in the dataset (those who are important in the largest number of rings regardless of ego and ring rank), in the case of soft clustering and hard clustering. The figure shows that soft clustering allows some topics to dominate the whole Journalists dataset. With soft clustering, topics 93, 51, 55, 95 and 72 are important for all six rings (the ego line is filled with colored squares) of

more than 50% of the ego networks. This, instead, is not the case when using hard clustering. The dominating topics in the case of soft clustering turn out being very generic ones. This is confirmed by looking at the most characteristic words in these topics in Table S3. For example topics 93 and 51, which were already among the most frequent in the hard cluster case are omnipresent in the soft cluster case, in addition to the topic 95 which is also generic but does not appear in the case of the hard cluster. We can therefore conclude that the price of a complete inclusion of tweets in our topic analysis through soft clustering only increases the noise level for all ego networks, materialized by a set of very generic topics that blur the real semantic characteristics of the rings. This is why we decided to put aside the results related to the soft clustering, in order to keep only the semantic distributions resulting from the hard clustering of HDBSCAN. Note that, in light of these results, the fact that we use only a small subset of available tweets does not impact on the relevance of our analysis. What we exclude are the tweets related to "noise" topics, in the sense that they are not able to strongly characterise the Twitter behaviour of users, and we focus only on tweets that are strongly belonging to topics, i.e., on the semantically characteristic part of users' Twitter activity. A consequence of this choice is that a tweet can only be associated to one topic with hard clustering. However, if HDBSCAN assigns a tweet as an outlier, it means that it is not close enough to any topic. Otherwise, it considers the dominant topic. Finding a way to perform a finer analysis of topics without adding too much noise is an interesting direction for future work.

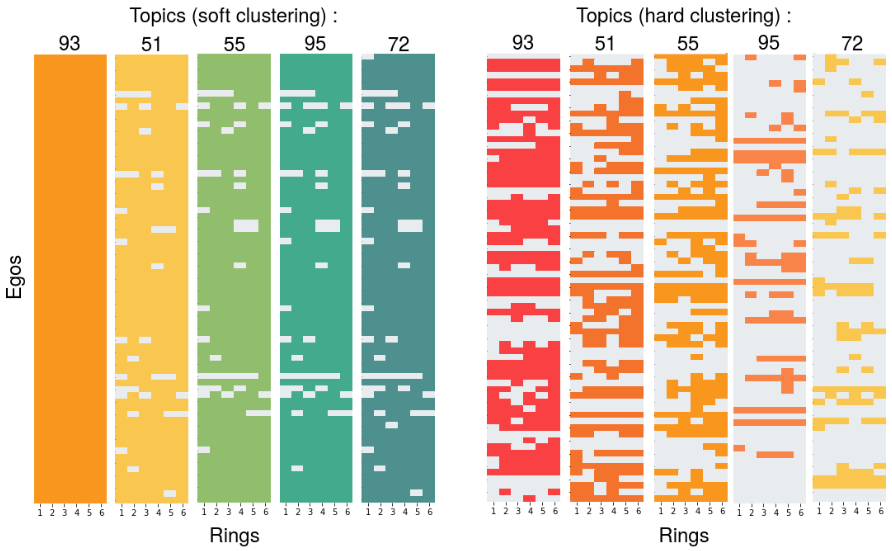

Fig. S2. Hard vs soft clustering: five most dominant topics. The two figures show how the five most important topics in the Journalists dataset are distributed, in the case of hard clustering (on the left) and soft clustering (on the right). For each topic, a grid is drawn in which the colored square means that the corresponding topic belongs to the most important topics of ring X of the ego network Y. Those topics are important for all six rings (the line is fully colored) for respectively 49%, 28%, 19%, 21%, 9% of all the ego networks of the dataset for the hard clustered configuration (left) and 100%, 75%, 75%, 74%, 68% for the soft clustered configuration.

S1.3 Additional tables

Table S1. Hashtags, links, emojis in the datasets. In the process of word extraction, the tweet is decomposed in tokens which are usually separated by spaces. These tokens generally corresponds to words, but they can also be links, emojis and others markers that are specific to the online language such as hashtags. The table gives the percentage of hashtags, links and emojis, which are tokens filtered out from the datasets.

	Percentage of hashtags	Percentage of links	Percentage of emojis
Journalists	1.34 %	7.27 %	0.20 %
Science writers	3.47 %	8.02 %	0.55 %
Random users #1	16.84 %	6.97 %	5.21 %
Random users #2	7.20 %	6.42 %	4.60 %

Table S2. Example of word extraction results.

Original tweet content	List of words after pre-processing
The @Patriots say they don't spy anymore. The @Eagles weren't taking any chances. They ran a "fake" practice before the #SuperBowl	spy, anymore, chance, run, fake, practice
#Paris attacks come 2 days before world leaders will meet in #Turkey for the G20. Will be a huge test for Turkey.	attack, come, day, world, leader, meet, huge, test, turkey
Latest garden species - the beautiful but destructive rosemary beetle, and a leafhopper (anyone know if this can be identified to species level from photo? Happy to give it a go) #30DaysWild #MyWildCity #gardening	late, garden, specie, beautiful, destructive, rosemary, beetle, leafhopper, know, identify, specie, level, photo, happy

Table S3. Most characteristic words per topic. They are obtained with a TF-IDF scoring.

Topic	Characteristic words (TF-IDF)
64	new obama administration tax white house comey donald president trump
24	cook lunch like dinner cheese chicken restaurant pizza food eat
93	boston read old like summer blue think google vega know
62	gop house obamacare vote repeal cut health senate republican tax
51	past february day tennis sentence week yesterday month ago year
93	boston read old like summer blue think google vega know
51	past february day tennis sentence week yesterday month ago year
55	london orleans nyc brooklyn statue monument time confederate new york
95	happy nice kind christmas great glad love thanks good thank
72	sharif judge state case pakistan gay execution supreme court arkansas

Table S4. Topics of the NYT journalists dataset. Most characteristic words and distribution in rings.

Topic	Characteristic words (TF-IDF)	R1	R2	R3	R4	R5	R6
0	australia australian story indigenous new	.006	.008	.009	.005	.005	.003
1	yankee baseball game pitch hit	.010	.007	.011	.011	.012	.011
2	italian soccer migrant libyan team	.009	.010	.004	.009	.004	.006
3	alabama governor senate robert moore	.000	.001	.001	.003	.004	.005
4	horse derby kentucky win race	.000	.002	.002	.003	.001	.004
5	apple mac use new silver	.001	.003	.009	.005	.005	.006
6	midwest south city today times	.013	.010	.010	.012	.014	.010
7	fox news pope fake vatican	.015	.009	.010	.006	.006	.008
8	french election macron pen paris	.000	.000	.008	.004	.002	.001
9	white shark nationalist president harvard	.002	.003	.003	.005	.003	.003
10	black slave african american asian	.014	.024	.021	.021	.022	.018
11	turkey turkish referendum protester president	.001	.003	.004	.001	.002	.001
12	cat mouse kitten game bureau	.001	.004	.006	.002	.004	.005
13	birthday happy halloween spring valentine	.002	.001	.001	.003	.000	.001
14	sleep bed nap asleep bedtime	.003	.004	.006	.007	.012	.007
15	phone sorry storm stuck quick	.006	.014	.006	.007	.010	.009
16	german right english angela fluent	.000	.001	.001	.000	.001	.001
17	football bowl super player anthem	.004	.007	.008	.005	.006	.007
18	brazil president brazilian scandal rio	.000	.003	.002	.003	.002	.004
19	flight plane fly helicopter passenger	.002	.002	.002	.001	.002	.002
20	beer vest clock declare power	.005	.006	.006	.009	.008	.005
21	dog pet puppy love good	.004	.005	.006	.006	.005	.005
22	wine red carpet school good	.007	.008	.008	.007	.006	.004
23	fish boat surf fishing sea	.000	.000	.002	.001	.005	.001
24	eat food pizza restaurant chicken	.001	.003	.004	.005	.008	.006
25	train subway station new delay	.000	.003	.001	.003	.002	.004
26	canada canadian refugee indigenous new	.001	.001	.002	.001	.001	.002
27	year minute yahoo day hour	.014	.013	.011	.009	.006	.005
28	bear montana wolf colorado wood	.003	.006	.003	.004	.004	.004
29	hockey game team stanley cup	.000	.002	.001	.000	.001	.000
30	snow ice winter cold arctic	.005	.005	.005	.003	.006	.008
31	texas special state education cap	.008	.004	.005	.007	.006	.006
32	sunday saturday night morning monday	.002	.002	.003	.003	.003	.004
33	friday thursday tuesday monday wednesday	.002	.003	.016	.006	.006	.007
34	moon space alien planet earth	.006	.004	.004	.006	.004	.005
35	japan abe japanese reactor scandal	.003	.005	.013	.008	.010	.011
36	china chinese hong new robot	.016	.007	.006	.006	.003	.007
37	north missile korean nuclear south	.000	.000	.002	.001	.001	.001
38	basketball league source trade season	.004	.006	.004	.002	.004	.003
39	sigh mike right wow know	.005	.009	.005	.004	.003	.003
40	twitter social medium like live	.003	.002	.003	.002	.002	.003
41	miss destroyer sailor collision ship	.041	.048	.041	.039	.055	.046
42	day july today year hour	.006	.006	.006	.007	.003	.003
43	movie watch film play episode	.001	.001	.001	.001	.001	.001
44	lobbyist intend dislike implication apology	.006	.016	.002	.002	.005	.002
45	california earthquake san francisco quake	.009	.003	.002	.001	.000	.000
46	hurricane florida irma storm harvey	.030	.046	.054	.069	.061	.066
47	prince woman crown ebony ballroom	.015	.007	.010	.001	.006	.004
48	iran iranian deal nuke president	.008	.004	.006	.005	.005	.005
49	syrian attack chemical strike weapon	.001	.003	.013	.008	.005	.010
50	russian russia trump investigation election	.001	.000	.002	.000	.002	.001

51	year ago month yesterday week	.009	.016	.013	.017	.010	.015
52	climate trump change paris cut	.001	.002	.001	.002	.008	.003
53	climate change oil paris carbon	.004	.002	.002	.001	.002	.002
54	tweet chronological good evergreen great	.038	.018	.037	.039	.030	.024
55	york new confederate time monument	.003	.002	.003	.003	.004	.008
56	tax estate cash bank fund	.001	.004	.001	.003	.003	.002
57	famine south yemen cholera venezuelan	.197	.147	.136	.121	.113	.127
58	listen book talk daily new	.022	.009	.003	.014	.013	.014
59	morning tomorrow good trial page	.007	.008	.006	.009	.011	.008
60	week hour month year marathon	.010	.008	.010	.007	.008	.010
61	million year billion spend marijuana	.004	.007	.006	.007	.007	.005
62	tax republican senate health cut	.023	.017	.017	.025	.031	.021
63	school high homework student college	.007	.008	.006	.006	.010	.008
64	trump president donald comey house	.003	.005	.003	.002	.002	.003
65	big palestinian time read story	.005	.002	.003	.006	.002	.005
66	fashion week mother wear model	.006	.004	.008	.005	.005	.006
67	dress leather pink skirt gown	.022	.022	.025	.026	.023	.029
68	tonight weekend atlanta bachelor georgia	.000	.001	.001	.001	.001	.001
69	song hip rap rock hop	.005	.001	.001	.000	.000	.001
70	broadway opera theater classical music	.006	.012	.002	.002	.002	.003
71	dot reporter peer time	.026	.017	.011	.021	.016	.017
72	arkansas court supreme execution gay	.006	.012	.010	.012	.009	.010
73	sexual harassment woman accuse allegation	.024	.034	.039	.024	.016	.025
74	wait bus happen mean depend	.038	.010	.007	.003	.003	.002
75	send address question shoot reach	.000	.000	.000	.001	.001	.001
76	car driver drive driving self	.001	.003	.001	.001	.002	.001
77	eclipse solar total delete totality	.029	.038	.040	.036	.037	.037
78	story news journalist accuse public	.070	.071	.084	.085	.115	.104
79	suicide trial roy conrad carter	.001	.003	.005	.005	.005	.006
80	die dead york robert roger	.001	.001	.005	.008	.004	.003
81	roe squeamish lisa susan collins	.002	.023	.005	.004	.005	.004
82	dislike unintended implication apology culture	.007	.012	.010	.011	.009	.008
83	lady girl yes elizabeth finale	.003	.004	.010	.008	.007	.007
84	book soon read write editor	.003	.000	.000	.000	.000	.002
85	best great video love game	.006	.012	.008	.010	.010	.012
86	bad terrible hate sorry awful	.002	.005	.008	.005	.005	.006
87	drug police arrest jail gang	.020	.016	.016	.014	.020	.020
88	kill militant police army congo	.000	.003	.007	.005	.003	.005
89	agree tweet important fascinate interesting	.001	.002	.002	.003	.002	.003
90	wrong bad argue moly mean	.003	.005	.011	.011	.007	.010
91	love woman genius happy sandra	.004	.005	.005	.005	.002	.004
92	yes true right joke correct	.002	.001	.002	.004	.004	.003
93	know vega google think blue	.011	.012	.011	.011	.015	.016
94	beautiful great cool gorgeous fun	.010	.012	.005	.004	.008	.006
95	good love glad great christmas	.024	.030	.015	.023	.019	.019
96	god know exactly gold yes	.016	.013	.012	.009	.010	.008
97	tho alex come like pat	.000	.002	.002	.003	.003	.002
98	kate congratulation diane karen welcome	.018	.012	.014	.028	.016	.017
99	read share contact matt paul	.003	.003	.003	.006	.003	.002

Table S5. Topics of the science writers dataset. Most characteristic words and distribution in rings.

Topic	Characteristic words (TF-IDF)	R1	R2	R3	R4	R5	R6
0	daily late luck today	.002	.002	.002	.003	.000	.003
1	baseball game lacrosse football player	.008	.008	.006	.007	.009	.011
2	follower week new canada right	.014	.013	.011	.017	.013	.016
3	video subtitle individual anonymous credit	.009	.005	.006	.002	.007	.005
4	aku morning good river countryside	.000	.000	.000	.000	.000	.000
5	aku morning good lake photo	.010	.004	.002	.004	.005	.002
6	badge earn level middle road	.009	.022	.017	.009	.006	.005
7	web nature post life plastic	.000	.000	.000	.000	.000	.000
8	essay environmental educator nature conservation	.009	.008	.004	.006	.007	.006
9	daily late clow soon hourly	.003	.003	.002	.005	.003	.003
10	submission album cheer shoot hello	.018	.008	.014	.012	.019	.023
11	submission album cheer shoot hello	.003	.008	.011	.007	.008	.008
12	poker play chess player best	.011	.006	.006	.006	.005	.006
13	robot human killer new job	.010	.008	.008	.005	.006	.007
14	year gorilla monkey story ape	.003	.008	.008	.007	.006	.007
15	white male quote diversity cause	.004	.010	.010	.016	.015	.016
16	christmas holiday year tree festive	.034	.049	.033	.045	.044	.056
17	plane flight fly spy airplane	.001	.005	.003	.005	.003	.004
18	eclipse space moon earth solar	.004	.002	.003	.002	.003	.003
19	african ancient beard genome revisit	.002	.003	.004	.008	.008	.005
20	air asthma pollution risk city	.003	.002	.002	.002	.002	.002
21	coffee shop drink caffeine cup	.024	.029	.022	.047	.029	.031
22	drink beer brewery beach ale	.001	.000	.000	.000	.000	.000
23	china chinese european scientific british	.003	.006	.003	.003	.002	.002
24	morning good perambulation wake bob	.003	.007	.009	.006	.011	.007
25	week virology new wildlife picture	.000	.000	.001	.000	.000	.001
26	negotiation britain tax british european	.013	.017	.007	.009	.005	.005
27	car driving self auto test	.046	.038	.038	.025	.027	.026
28	happy birthday year mother wedding	.004	.004	.008	.005	.004	.008
29	twitter mention reach social medium	.003	.004	.003	.003	.004	.003
30	apple mobile search phone new	.008	.005	.006	.007	.005	.009
31	weekly microbiology science episode new	.000	.001	.000	.001	.000	.000
32	social medium fake news combat	.011	.012	.011	.008	.009	.007
33	record hot year high warm	.039	.029	.043	.056	.050	.056
34	journalist join hear sally tonight	.007	.010	.007	.012	.009	.011
35	prize chemistry win medicine physiology	.006	.005	.008	.005	.005	.006
36	chicken meat eat animal barn	.000	.004	.003	.007	.004	.004
37	sleep bed night nap dream	.034	.036	.013	.015	.015	.018
38	earthquake quake tsunami seismic big	.003	.006	.009	.005	.005	.005
39	ice arctic winter snow antarctica	.005	.011	.009	.007	.007	.008
40	canada canadian maple citizenship government	.005	.005	.012	.011	.006	.009
41	california wildfire northern burn flee	.004	.013	.006	.005	.009	.008
42	hurricane storm flood rain irma	.006	.008	.012	.009	.009	.010
43	old fossil human year ancient	.001	.004	.005	.004	.007	.005
44	frog otter snake amphibian rid	.069	.067	.071	.067	.076	.075
45	pterosaur skull crest cornified animal	.005	.002	.003	.004	.003	.001
46	bird spider bat flower moth	.004	.009	.014	.011	.012	.015
47	dinosaur fossil bird mammal discover	.119	.107	.146	.137	.148	.142
48	shark whale sea fish ocean	.002	.002	.005	.004	.004	.006
49	bear wolf polar kill rhino	.006	.007	.008	.008	.010	.011
50	dog puppy good breed love	.002	.002	.003	.002	.001	.001

51	chocolate eat pizza pie cheese	.002	.003	.003	.002	.002	.003
52	food delicious fortune restaurant love	.009	.002	.003	.003	.002	.002
53	cat dog kitten like think	.037	.039	.032	.032	.035	.035
54	rule tobacco regulatory million health	.000	.000	.000	.000	.000	.000
55	year time hour paper china	.008	.007	.007	.007	.004	.002
56	woman award stem girl winner	.001	.002	.003	.003	.002	.004
57	editor story wired write business	.041	.033	.043	.044	.038	.041
58	car bicycle bike crash driving	.001	.002	.002	.001	.004	.002
59	solar power wind energy electricity	.000	.000	.000	.000	.000	.000
60	american america black prescription slavery	.001	.001	.005	.003	.004	.006
61	die child woman bad parent	.020	.011	.008	.009	.013	.011
62	health medical care patient doctor	.009	.005	.014	.007	.007	.010
63	photo pic sharpen color apply	.004	.005	.007	.007	.007	.008
64	cancer new cell mouse disease	.004	.002	.001	.003	.008	.005
65	chromosome human horse embryo gene	.003	.005	.006	.004	.003	.003
66	republican senate house senator white	.017	.008	.008	.009	.007	.006
67	year day week halloween time	.002	.000	.000	.002	.001	.000
68	trump administration president climate donald	.002	.005	.002	.004	.003	.003
69	nuclear north weapon war iran	.003	.006	.001	.003	.003	.002
70	coal oil climate fuel kentucky	.004	.004	.012	.005	.012	.006
71	climate change carbon scientist report	.007	.003	.003	.003	.004	.005
72	defense arrive plant episode week	.001	.008	.009	.005	.007	.006
73	kill police murder arrest officer	.010	.009	.011	.012	.009	.007
74	documentary film watch new series	.013	.014	.003	.005	.002	.001
75	year end hour ago chronicle	.000	.000	.000	.000	.000	.000
76	send address dot touch chat	.004	.002	.001	.003	.002	.004
77	science donation match great recur	.009	.003	.004	.005	.006	.008
78	like good way think know	.001	.004	.002	.003	.003	.001
79	boston stereo arena queen wed	.021	.016	.008	.010	.009	.008
80	great year sing night happy	.000	.001	.007	.003	.006	.001
81	night stream miss catch tonight	.005	.004	.006	.005	.005	.004
82	week month year new tomorrow	.001	.005	.005	.004	.004	.003
83	science student school week scientist	.006	.020	.011	.013	.019	.010
84	sunday saturday night come need	.053	.041	.044	.032	.037	.029
85	thursday friday join wednesday tuesday	.001	.001	.002	.002	.002	.002
86	science sexual harassment obituary journalism	.011	.011	.005	.008	.007	.006
87	community follow rank step work	.010	.007	.012	.010	.009	.012
88	free article site tweet want	.011	.009	.008	.006	.009	.006
89	book read weekend science journal	.000	.000	.000	.000	.000	.000
90	mean think worry thing point	.000	.004	.001	.002	.004	.004
91	know right sure check want	.012	.020	.008	.010	.006	.009
92	bad people medium crazy like	.003	.001	.001	.001	.001	.001
93	sorry bad terrible sad weird	.038	.023	.023	.031	.021	.020
94	god nope test idea know	.015	.014	.011	.009	.012	.009
95	yes agree wow mean whoa	.005	.007	.008	.012	.009	.011
96	glad kind great love enjoy	.000	.004	.003	.006	.001	.001
97	good awesome love cool nice	.002	.001	.003	.002	.003	.002
98	fan week big congratulation mull	.000	.000	.000	.000	.000	.000
99	bless andy congratulation paul mate	.003	.003	.004	.003	.003	.002

Table S6. Topics of the random users #1 dataset. Most characteristic words and distribution in rings.

Topic	Characteristic words (TF-IDF)	R1	R2	R3	R4	R5	R6
0	twitter mention reach week like	.003	.002	.002	.002	.003	.002
1	automatically unfollowed check follow people	.005	.004	.008	.004	.005	.004
2	natural naturally soon tune launch	.004	.005	.005	.004	.005	.005
3	post photo atlantic raw valley	.011	.012	.012	.015	.017	.017
4	week fan big boy great	.001	.001	.001	.001	.001	.001
5	replacement screen ram core battery	.005	.004	.005	.005	.005	.006
6	practice spanish read news post	.007	.007	.007	.007	.008	.008
7	bristol story chronicle daily include	.001	.001	.001	.001	.001	.001
8	cannabis marijuana medical weed industry	.002	.002	.002	.003	.004	.003
9	australia visa immigration australian apply	.006	.002	.002	.001	.001	.001
10	alert trance dance hit triple	.007	.007	.007	.005	.005	.005
11	hire job post pro apply	.000	.002	.001	.002	.004	.004
12	music available game prophesy gospel	.000	.002	.002	.002	.002	.002
13	happy peep good thanksgiving holiday	.022	.025	.029	.028	.026	.025
14	canada immigration apply express entry	.011	.013	.006	.004	.005	.006
15	visit information weekly clue chat	.001	.003	.001	.001	.001	.001
16	track rock follower today outlaw	.001	.002	.002	.003	.004	.004
17	late daily innovative horse source	.005	.004	.004	.005	.004	.004
18	moon space mar astronaut mission	.009	.004	.004	.002	.002	.001
19	road gold world win champ	.021	.017	.013	.012	.008	.009
20	link subscribe click channel registration	.003	.005	.005	.005	.004	.005
21	red blue sugar mug titan	.003	.003	.004	.004	.005	.004
22	catholic priest pope church prayer	.000	.000	.001	.001	.001	.001
23	trading risky suitable net close	.003	.002	.003	.003	.003	.005
24	life sunday breath coach insurance	.004	.007	.005	.005	.005	.004
25	god lord jesus christ unto	.005	.003	.003	.003	.002	.002
26	associate page log principal excerpt	.001	.002	.003	.002	.002	.003
27	amazon offer bank discount author	.029	.020	.017	.012	.014	.011
28	phone car tune today mobile	.006	.003	.007	.008	.009	.011
29	car hire plate vat drive	.006	.008	.011	.007	.008	.009
30	christmas merry gift festive day	.001	.003	.003	.003	.005	.005
31	friday weekend happy day halloween	.001	.001	.001	.001	.002	.002
32	tea beer drink come brewery	.001	.001	.001	.001	.002	.001
33	yoga teacher japanese meditation training	.006	.006	.007	.006	.007	.006
34	life weight lose people think	.017	.016	.016	.018	.018	.019
35	black white american fear legging	.008	.010	.009	.008	.009	.008
36	today evangelist shower angela help	.009	.009	.012	.013	.013	.014
37	thing dream life right time	.020	.028	.026	.027	.029	.029
38	monday week morning happy good	.004	.006	.009	.007	.007	.007
39	coffee cup morning good day	.009	.017	.009	.007	.005	.005
40	password best wednesday frustration day	.004	.003	.004	.004	.004	.003
41	dog pet puppy love dane	.006	.003	.004	.004	.005	.004
42	cat kitten home lover happy	.009	.008	.011	.013	.013	.014
43	apply badge level earn job	.019	.006	.006	.004	.003	.003
44	tuesday today day good life	.004	.006	.005	.006	.005	.005
45	food breakfast eat recipe chris	.013	.017	.015	.019	.022	.021
46	cake chocolate cream ice birthday	.006	.006	.009	.006	.006	.008
47	look nice delicious yummy forward	.006	.005	.008	.008	.009	.011
48	flight dana fly update gate	.009	.008	.009	.010	.008	.010
49	chicken curry lunch green menu	.005	.005	.003	.004	.003	.004
50	follow hey kindly smile fib	.010	.009	.011	.013	.011	.010

51	bedroom home house pool village	.031	.032	.036	.035	.034	.034
52	shop fashion dress wedding buy	.008	.008	.006	.005	.007	.005
53	cricket win match wicket cup	.005	.004	.004	.005	.004	.004
54	win rocket game final score	.001	.001	.001	.001	.001	.001
55	basketball football team game soccer	.055	.051	.056	.055	.053	.057
56	beautiful cute hope bird look	.004	.001	.001	.001	.001	.001
57	sorry inconvenience contact hear team	.006	.006	.008	.008	.006	.007
58	sleep bed night wake nap	.005	.006	.006	.006	.007	.005
59	winter snow cold ski rain	.002	.001	.002	.003	.002	.002
60	tonight winner night win ticket	.013	.011	.008	.009	.006	.006
61	connect let follow group family	.008	.009	.009	.010	.011	.011
62	social medium hilarious engagement marketing	.010	.004	.005	.003	.002	.002
63	live music official video bad	.022	.024	.025	.027	.029	.025
64	dance befit class studio join	.001	.001	.001	.002	.002	.002
65	video learn color alphabet child	.006	.006	.006	.006	.005	.006
66	content write writer currently start	.011	.010	.009	.009	.009	.007
67	climate east change late south	.057	.038	.036	.033	.033	.035
68	stay park hostel hotel board	.015	.022	.019	.024	.020	.019
69	oil climate join fossil fuel	.089	.103	.104	.106	.098	.093
70	birthday happy wish bless year	.009	.006	.009	.006	.007	.006
71	morning good golf bless day	.008	.009	.008	.008	.010	.008
72	address send hello look certainly	.001	.001	.001	.001	.001	.002
73	staff dudley health nurse mental	.008	.010	.011	.011	.010	.011
74	vulnerable rat outstanding agency child	.002	.002	.004	.005	.007	.006
75	help miss autism interested locate	.004	.006	.003	.003	.003	.003
76	tutor tip directory literacy foot	.003	.001	.001	.002	.001	.001
77	cancer patient therapy cell treatment	.006	.003	.000	.000	.000	.000
78	west movie blast film watch	.023	.022	.026	.026	.026	.024
79	million year store billion investment	.001	.002	.001	.001	.001	.001
80	new salary happy year profile	.002	.001	.002	.003	.002	.002
81	appreciate share shout homeless tweet	.003	.002	.002	.002	.002	.002
82	school exam dismissal free generate	.016	.021	.019	.019	.018	.017
83	mother brother son queen love	.011	.012	.013	.015	.016	.017
84	book savvy silly society update	.004	.004	.004	.004	.005	.005
85	woman ass sexy sensual sophisticated	.015	.018	.018	.018	.014	.015
86	day verse valentine great grateful	.002	.002	.003	.003	.001	.002
87	cloud marketing digital network robot	.106	.105	.104	.107	.111	.111
88	career business support information employer	.001	.003	.002	.002	.001	.002
89	year wait month code week	.006	.009	.007	.005	.005	.004
90	welcome sacrifice salute nancy champagne	.009	.010	.014	.015	.015	.016
91	love congratulation feedback great hug	.003	.005	.005	.004	.004	.004
92	creation awesome create think look	.018	.019	.020	.021	.024	.025
93	india anniversary indian birth kashmiri	.017	.013	.009	.008	.007	.008
94	amen preach word naa ouch	.004	.004	.005	.005	.006	.006
95	dream true agree old believe	.003	.003	.003	.005	.005	.004
96	vote know people hold yes	.006	.009	.008	.008	.011	.012
97	trump president russia hillary lawyer	.007	.004	.007	.006	.006	.005
98	arrest police man kill murder	.000	.001	.001	.002	.001	.002
99	bad sad disrespectful awful disgust	.000	.000	.000	.000	.000	.000

Table S7. Topics of the random users #2 dataset. Most characteristic words and distribution in rings.

Topic	Characteristic words (TF-IDF)	R1	R2	R3	R4	R5	R6
0	temp sea pressure rain weather	.008	.009	.012	.012	.011	.011
1	job check nurse advisor ref	.002	.004	.006	.003	.006	.006
2	data storage file holiday song	.004	.007	.011	.007	.006	.007
3	morning good kevin steve vacancy	.019	.013	.015	.018	.014	.014
4	live saturday stream masquerade laugh	.011	.010	.009	.008	.007	.004
5	jump long pit radio runway	.002	.005	.004	.005	.002	.002
6	pitch synthetic turf artificial sport	.006	.008	.006	.008	.009	.007
7	market sign september risk easy	.000	.000	.000	.000	.000	.000
8	consultant resin sport pitch flooring	.009	.012	.013	.008	.007	.008
9	aquarius sensational today seventy happen	.017	.021	.019	.016	.019	.022
10	playground marking key stage game	.012	.013	.009	.017	.011	.013
11	gallery collection art contemporary home	.006	.002	.002	.003	.002	.002
12	cancer mouth breast research today	.005	.008	.010	.007	.008	.008
13	safety train air cylinder pneumatic	.019	.021	.019	.020	.020	.017
14	trade wale choose big car	.006	.005	.004	.005	.004	.006
15	beautiful cute look amaze adorable	.066	.057	.063	.077	.072	.075
16	manager director yoga technical executive	.008	.011	.007	.006	.008	.011
17	course training certificate lunch click	.005	.007	.004	.004	.004	.005
18	news north northern west warrior	.040	.042	.045	.045	.050	.043
19	mobility product salary showroom look	.002	.002	.002	.002	.003	.003
20	china chinese outbreak congo measles	.022	.021	.022	.023	.023	.021
21	ref level surfacing sale representative	.009	.010	.013	.009	.009	.007
22	interview job excellent benefit tip	.001	.002	.003	.003	.002	.002
23	truck law year new minute	.015	.017	.013	.011	.008	.008
24	privacy place data speaker security	.011	.009	.010	.011	.012	.011
25	rule entry voucher submit year	.007	.008	.007	.005	.007	.006
26	late daily predator bullet pip	.001	.002	.002	.003	.003	.003
27	twitter mention reach week like	.019	.018	.020	.021	.022	.018
28	birthday happy hope soon wish	.003	.003	.004	.005	.005	.006
29	cheer agree true baby mate	.004	.005	.004	.004	.004	.004
30	hockey court tennis final surface	.003	.002	.002	.002	.004	.002
31	free instant horse audit tip	.001	.000	.000	.000	.001	.000
32	branch rate store available buy	.000	.000	.000	.000	.000	.000
33	support help child people sport	.011	.017	.013	.010	.014	.014
34	tropical storm thunderstorm weather rain	.010	.009	.009	.010	.012	.009
35	sleep bed night nap asleep	.003	.002	.002	.004	.002	.002
36	property bedroom tax station family	.018	.011	.005	.004	.004	.005
37	ship cruise new marine boat	.019	.028	.029	.033	.037	.033
38	new star review unit charge	.003	.004	.003	.003	.003	.004
39	wine competition enter medal sommelier	.004	.004	.003	.006	.004	.005
40	cost value low decision help	.007	.007	.008	.010	.009	.007
41	coffee tea cup lunch grandma	.003	.004	.003	.004	.004	.005
42	christmas merry gift year festive	.047	.041	.052	.054	.042	.051
43	click link workshop business poetry	.040	.040	.043	.040	.046	.042
44	night tonight bar drink beer	.006	.005	.006	.006	.007	.006
45	number guide model mary information	.033	.022	.015	.016	.010	.012
46	cat kitten bruce love like	.049	.037	.053	.052	.054	.057
47	food farm production course eat	.002	.005	.001	.001	.002	.001
48	attack data breach security user	.024	.039	.043	.041	.038	.035
49	garden summer plant grow flower	.005	.004	.005	.007	.008	.008

50	dog puppy pet guide animal	.007	.006	.003	.004	.003	.005
51	pizza chicken cheese meat sausage	.004	.005	.005	.004	.005	.004
52	miss today gemini watch courtesy	.007	.004	.007	.008	.006	.007
53	health mental cigarette tobacco cricket	.001	.001	.002	.002	.001	.001
54	brain injury scientist researcher science	.000	.002	.001	.000	.001	.001
55	climate green change carbon environmental	.022	.009	.009	.005	.007	.006
56	fisherman fish beanie fishery marine	.005	.001	.002	.001	.002	.001
57	photo learn range support publish	.015	.018	.018	.016	.019	.017
58	follow automatically unfollowed check person	.001	.003	.002	.002	.002	.002
59	movie game best funny hot	.001	.000	.001	.001	.001	.000
60	happy car customer new trade	.004	.004	.004	.006	.005	.005
61	shower black halloween dance look	.010	.011	.010	.010	.012	.013
62	ray order edition release win	.026	.029	.028	.030	.030	.035
63	tomorrow evening close support message	.006	.005	.005	.007	.004	.005
64	ticket tour sale wait announce	.002	.006	.008	.007	.006	.006
65	music album new single pic	.011	.011	.010	.011	.012	.013
66	monday wednesday tuesday flight fly	.021	.018	.019	.022	.019	.023
67	friday library thursday fact hub	.005	.008	.009	.007	.008	.007
68	pisces scorpio virgo aries stop	.004	.005	.004	.004	.005	.006
69	rugby story world news cup	.012	.008	.007	.006	.007	.007
70	red player play win game	.003	.003	.004	.002	.002	.002
71	football league weekend win round	.010	.011	.012	.012	.013	.014
72	model age commercial shoot female	.014	.013	.008	.005	.004	.004
73	trump donald president like america	.002	.005	.006	.006	.008	.008
74	school bullying start change cover	.005	.010	.009	.007	.010	.009
75	student need require math support	.002	.003	.002	.002	.002	.003
76	congratulation award woman queen category	.006	.007	.007	.008	.007	.006
77	sorry order address number hear	.005	.002	.002	.002	.001	.001
78	update android store form creator	.005	.005	.005	.005	.008	.006
79	day valentine today good happy	.019	.014	.013	.012	.013	.010
80	cement airport retail duty travel	.002	.002	.002	.002	.003	.002
81	month year week contract sunday	.006	.012	.009	.008	.007	.005
82	business parent feature social medium	.003	.004	.003	.002	.002	.002
83	bank payment launch platform banking	.009	.008	.006	.006	.007	.006
84	week hour month image shot	.017	.017	.015	.020	.016	.018
85	leadership development network skill leader	.003	.005	.005	.005	.005	.003
86	hug paw love send david	.003	.005	.003	.003	.004	.003
87	police man old jail arrest	.012	.012	.010	.010	.010	.010
88	road car driver vehicle cyclist	.012	.010	.008	.008	.008	.008
89	car race drive raceway driver	.017	.014	.013	.009	.011	.006
90	vote boris deal labour party	.001	.002	.002	.002	.003	.004
91	tip time management try start	.006	.010	.011	.007	.009	.016
92	address send password congratulation number	.007	.010	.009	.007	.007	.008
93	bad hate sad sorry wrong	.004	.005	.005	.005	.006	.006
94	subscription address sorry hear look	.001	.001	.002	.001	.001	.002
95	echo team sorry touch order	.003	.002	.002	.002	.004	.004
96	dont think know game like	.011	.011	.005	.006	.004	.005
97	love sun island change book	.002	.003	.003	.004	.003	.005
98	big follower fan week share	.010	.005	.006	.006	.007	.007
99	yes yeah xmas amen everyday	.007	.005	.009	.007	.006	.004

References

1. Boldrini C, Toprak M, Conti M, Passarella A. Twitter and the press: an ego-centred analysis. In: Companion Proceedings of the The Web Conference'18; 2018. p. 1471–1478.
